# FOCUS harmonic scalpel compared to conventional hemostasis in open total thyroidectomy - a prospective randomized study

**DOI:** 10.1186/1916-0216-42-62

**Published:** 2013-12-20

**Authors:** Yun-fei Duan, Wei Xue, Feng Zhu, Dong-lin Sun

**Affiliations:** 1 Department of General Surgery, The Third Affiliated Hospital of Soochow University, 185 Juqian Street, Changzhou, Jiangsu 213003, China

**Keywords:** Thyroid surgery, Total thyroidectomy, Harmonic scalpel, Hemostasis, Hypoparathyroidism, Hypocalcemia

## Abstract

**Background:**

Hemostasis in thyroid surgery is of utmost importance for a successful surgery and an uneventful postoperative course. Our aim was to evaluate the effectiveness of the FOCUS Harmonic Scalpel in patients undergoing open total thyroidectomy.

**Methods:**

In this study, 778 patients were randomized into 2 groups based on the surgical technique used: group I comprised the conventional clamp-and-tie technique, group II comprised patients in whom the FOCUS Harmonic Scalpel was used exclusively. The groups were compared in regard to surgical time, complications, and hospital stay.

**Results:**

Surgical time was significantly lower in group II compared with group I (79 ± 21.5 min vs.125 ± 30.4, respectively, P < 0.001). Twenty-seven patients (6.94%) in group I experienced symptomatic hypocalcemia requiring calcium and/or vitamin D therapy versus 14 patients (3.6%) in group II, with statistically significant difference (P < 0.05). Mean post-operative hospital stay was significantly lower in group II compared with group I (2.6 ± 0.9 vs. 2.9 ± 1.0; P < 0.001).

**Conclusions:**

The FOCUS Harmonic Scalpel can shorten operative time and hospital stay, reduce incidence of symptomatic hypocalcemia but not transient hypoparathyroidism, and show no significance on recurrent nerve injury. FOCUS Harmonic Scalpel is supposed to be a more reliable and safe instrument that can take place of the clamp-and-tie technique in total thyroidectomy.

## Introduction

Since Kocher and Billroth first described an acceptable technique of a standardized thyroid surgery in the late 19th century [1], thyroidectomy has become one of the most frequent surgery, and bilateral total thyroidectomy (TT) is performed in the majority of thyroid diseases.

Hemostasis during thyroidectomy can be performed by classic suture ligation with clamp-and-tie maneuvers and/or by electrocoagulation. Whereas suture ligation is a time consuming procedure and carries the risk of knot slipping; electrocautery on the other hand is an unattractive alternative because it implies the potential risk of injuring surrounding tissues from dispersion of heat [2].

A technical advance in the early 1990s was the development of an ultrasonically activated device that includes shears and a scalpel, thus permitting the surgeon to cut tissues and control blood loss at the same time [3]. The FOCUS Harmonic Scalpel uses high-frequency mechanical energy to cut and coagulate tissues and vessels simultaneously without the need for knot tying [4]. Some reports on the use of the harmonic scalpel were encouraging, showing a significant reduction in surgical time [5]. In this prospective randomised study, we evaluated the efficiency, safety, and the impact on the surgical outcome of the utilisation of the FOCUS Harmonic Scalpel during CT.

## Materials and methods

**Figure 1:**
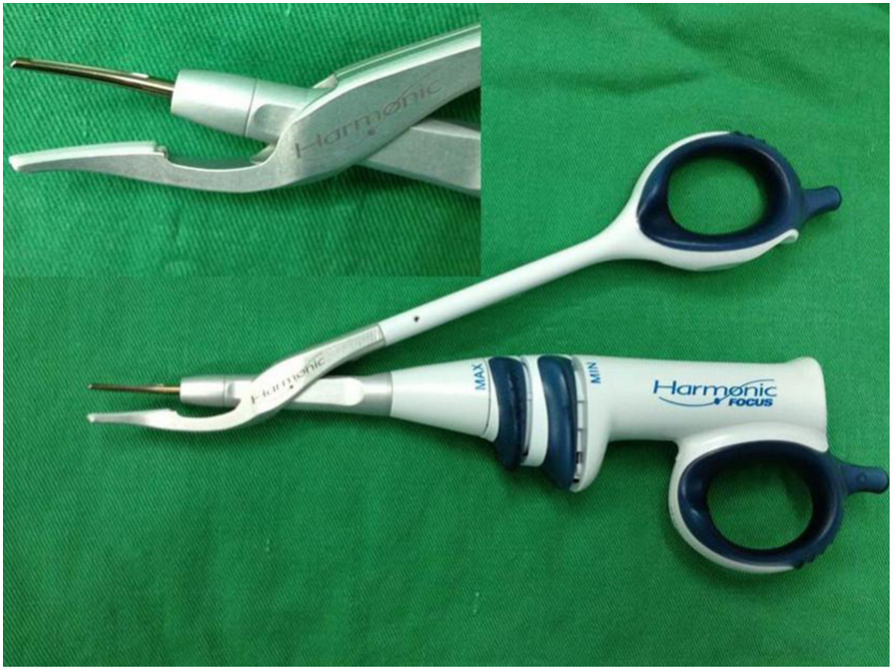
Focus Harmonic Scalpel (Ethicon Endo-Surgery, Inc, Cincinnati, Ohio, USA).

Between January 2009 and December 2012, 778 patients scheduled for open TT consented to be included in the study and were randomly assigned to TT with the use of FOCUS Harmonic Scalpel (Figure 1) (group I) or with the clamp-and-tie technique (group II). The protocol of this study have been submitted and approved by the ethical committee of our Institution. All the patients gave their informed written consent to be included in this study.

### Study design

All the patients scheduled to undergo TT were considered eligible to be included in the study in absence of exclusion criteria. Exclusion criteria were age <18 years, previous neck surgery or irradiation, eligibility for minimal invasive video-assisted thyroidectomy, need for central and/or lateral lymph node dissection and concomitant parathyroid disorders.

Patients were informed about the study at time of hospitalization by a staff surgeon. They were enrolled after accepting to participate in the study and signing the informed consent. A specific questionnaire concerning demographic and preoperative clinical patient’s characteristics was administered before surgery. At the time of recruitment, three additional questionnaires were consigned to patients for postoperative evaluation, to be fulfilled at discharge, 1 and 3 months after the operation, respectively.

At time of surgery, patients were randomly assigned to undergo TT with or without the utilisation of the FOCUS Harmonic Scalpel (group I and group II, respectively). All the patients were blinded about the technique used for hemostasis. Our medical staff was responsible for the collection of the clinical data, including preoperative diagnosis, operative time (from skin incision to skin closure), operative procedure, postoperative complications, hospital stay and final histology.

Laryngoscopy to check vocal cord motility was performed preoperatively only in symptomatic patients and postoperatively in all the cases. Postoperative calcium and parathyroid hormone (PTH) levels were monitored in all the patients. Hypocalcemia was defined as a serum calcium level below 2.0 mmol/l. Hypocalcemic patients received supplementation therapy even if asymptomatic. Supplementation therapy included always oral calcium and/or vitamin D (calcitriol). Supplementation therapy was subsequently tapered on the basis of serum calcium measurements. Recurrent laryngeal nerve palsy and hypoparathyroidism were defined definitive if they did not recover within 12 months after the operation.

Follow-up evaluation was obtained by outpatient consultation or telephone contact 1 and 3 months after surgery.

### Surgical technique

**Figure 2:**
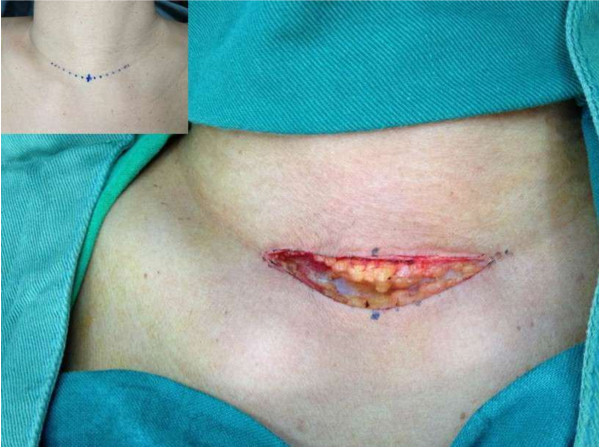
A 4 to 6 cm low collar incision.

**Figure 3:**
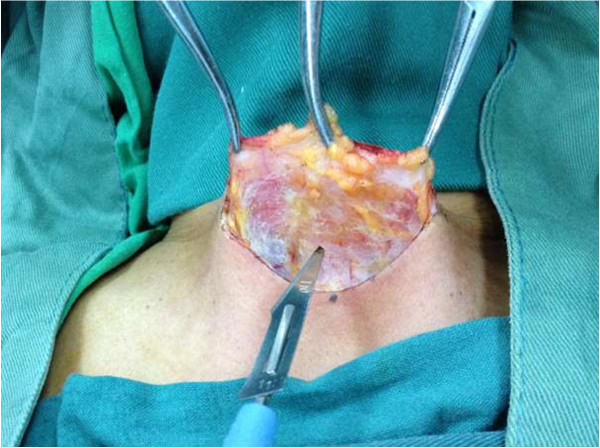
Develop subplatysmal flaps with high frequency electric knife.

A TT for benign or malignant thyroid disease was performed under general anesthesia and with endotracheal intubation in all cases. All patients were positioned, monitored, and draped in the standard way. A 4 to 6 cm low collar incision was made (Figure 2). Subplatysmal flaps were developed with high frequency electric knife and the strap muscles were separated at the midline (Figure 3). When the gland was exposed, the thyroid vessels were ligated in one of the following ways.

### The conventional clamp-and-tie technique

Formal thyroid vessels (inferior middle and superior) were clamped and tied using 3/0 silk sutures, and in all other vessels absorbable 4/0 sutures and/or electrocauterization were used.

### The use of the FOCUS for all thyroid vessels

**Figure 4:**
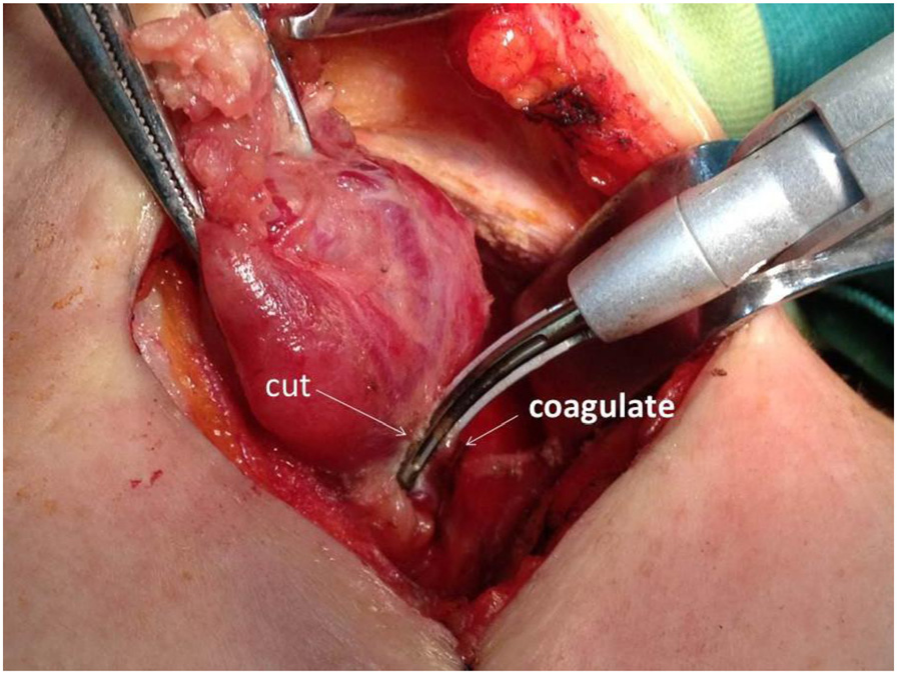
**Ligation of the inferior thyroid vessels with a double-ligation technique.** In the distal part of the artery we used the device just to coagulate the vessel. In the proximal part, after coagulation, we cut the tissue.

All thyroid vessels were ligated with the FOCUS Harmonic Scalpel. For the ligation of the superior and inferior thyroid vessels, we used a double-ligation technique in which the device was used in 2 succeeding areas of the vessel. In the distal part of the artery we used the device just to coagulate the vessel. In the proximal part, after coagulation, we cut the tissue (Figure 4). No suture ligation was used.

**Figure 5:**
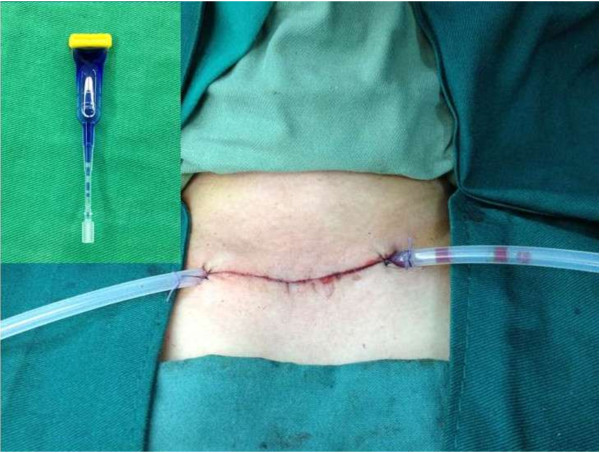
Close incisions of skin with tissue adhesive Histoacryl Blue.

Further surgical steps were similar for group I and group II. Recurrent laryngeal nerves and parathyroid glands were always identified. In all patients a closed suction drain was placed below the strap muscles. The drains were removed 24–48 hours after surgery. The wound was closed using interrupted 4/0 absorbable sutures (Vycril, Ethicon) for the strap muscles and platysma. Incisions of skin were closed without suturing by using tissue adhesive Histoacryl Blue (B. Braun Meisungen AG, Melsungen, Germany) (Figure 5).

### Statistical analysis

Data were analyzed with the use of the SPSS software (Version16; SPSS Inc., Chicago, IL, USA). Statistical analysis was performed using Student’s *t* test for continuous variables and Fisher exact test for categorical variables. The results were expressed as mean ± SD if not stated otherwise. All tests were double-sided and the level of statistical significance was set at a P value of less than 0.05.

## Results

The two groups did not differ in regard to age, sex, and pathologies. The demographics and diagnosis of each group were shown in Table 1.

**Table 1:** Patient’ demographics and diagnosis

	**Group I (n = 389)**	**Group II (n = 389)**	**P value**
Mean age, y (mean ± SD)	50.1 ± 19.3	48.5 ± 21.8	NS
Sex (F/M)	323/66	332/57	NS
Diagnosis:			
Multinodular goiters	313	309	NS
Hashimoto’s thyroiditis	9	7	NS
Thyroid cancer	56	64	NS
Graves disease	11	9	NS

*F* female, *M* male, *SD* stand deviation, *NS* not significant.

Surgical time was significantly lower in group II compared with group I (79 ± 21.5 min vs.125 ± 30.4, respectively, P < 0.001) (Table 2).

**Table 2:** Comparison of the operative and post-operative results of patients

	**Group I (n = 389)**	**Group II (n = 389)**	**P value**
Surgical time, min(mean ± SD)	125 ± 30.4	79 ± 21.5	<0.001
PTH, pmol/l (mean ± SD)	2.64 ±1.09	2.75 ± 1.22	NS
Calcium, mmol/l (mean ± SD)	2.07 ± 0.29	2.11 ± 0.34	NS
Transient hypoparathyroidism	40	36	NS
Symptomatic hypocalcemia	27	14	<0.05
Hemorrhage (re-operation)	1	0	NS
Transient recurrent nerve palsy	1	0	NS
Hospital stay, d(mean ± SD)	2.9 ± 1.0	2.6 ± 0.9	<0.001

*SD* stand deviation, *NS* not significant.

In group I mean PTH level at first post-operative day was 2.64 ± 1.09 pmol/l; mean calcium level at first post-operative day was 2.07 ± 0.29 mmol/l. In group II mean values were 2.75 ± 1.22 pmol/l and 2.11 ± 0.34 mmol/l, respectively. These differences were not statistically significant. There was no significant difference between the two groups concerning transient hypoparathyroidism: 40 patients (10.28%) in group I versus 36 (9.25%) in group II, P > 0.05. However, 27 patients (6.94%) in group I experienced symptomatic hypocalcemia requiring calcium and/or vitamin D therapy versus 14 patients (3.6%) in group II, with statistically significant difference (P < 0.05) (Table 2). In these patients, discharge was postponed until the restoration of the serum calcium levels to a normal range (2.0–2.9 mmol/l).

There was one patient with clinically evident recurrent nerve palsy was observed in group I, who recovered 6 months after surgery. One patient with cervical hematomas required re-exploration within 24 hours. No other postoperative complications were observed. Mean post-operative hospital stay was significantly lower in group II compared with group I (2.6 ± 0.9 vs. 2.9 ± 1.0; P < 0.001) (Table 2).

## Discussion

The thyroid gland has an extensive vascular network, therefore it is of primary importance to achieve good hemostasis to avoid postoperative hemorrhage which potentially causes patient’s asphyxia and death [6].

Total thyroidectomy requires massive clamp-and-tie maneuvers for the small thyroid vessels, the use of conventional technique is time consuming. Other common instruments available for thyroid surgery are mono or bipolar diathermy, which are unsafe in total thyroidectomy because of the risk of damaging the adjacent structures from lateral thermal spread.

FOCUS Harmonic scalpel is the first device specifically designed for every procedure where meticulous dissection and effective hemostasis is of paramount importance, particularly in a narrow operating field as in thyroid surgery. It is characterized by lesser weight and smaller size compared to other shears; the thin and curved tip, associated to the ergonomic shape and consequent handiness, guarantees precise and safe dissection. Its specifications permit the safe ligation of vessels less than 3 mm in diameter. However, some reports that suggested the use of the harmonic scalpel for larger vessels such as the superior thyroid artery [7]. Siperstein et al [8] suggested the use of the ultracision for all thyroid vessels and were the first to describe the double-ligation technique. The key point for hemostasis with the use of the double-ligation technique is that the vessels should be coagulated in 2 succeeding areas, which is similar to the one we used in group II.

In our study, the use of the FOCUS was associated to a statistically significant reduction in surgical time, with a mean advantage of 46 min for each surgery. Hemostasis with FOCUS is quick and effective, and we did not observe any post-operative hemorrhage in group II.

In addition, meticulous hemostasis is essential to ensure a clear surgical field and to avoid inadvertent damage to adjacent vital structures, e.g., to the superior and the recurrent laryngeal nerves and the parathyroid glands, as the two most significant complications with incidences regarding permanent recurrent laryngeal nerve palsy and hypoparathyroidism of up to 14 percent and 4 percent, respectively [9]. To enhance safety, the FOCUS hand piece ends in 2 jaws: an active stable jaw for the transmission of energy and a movable jaw that is used for tissue clamping. The energy is not transmitted to the movable part, which can therefore be used as a barrier against thermal transmission [10]. To minimize this risk, we used cold-wet gauze absorb heat during coagulation near the nerve, reducing the local temperature and consequently potential thermal damages. In our group II, there was no recurrent nerve palsy occur.

In our series there were no statistically significant differences in PTH level and calcium level at first post-operative day between group I and group II, which were considered as effective parameters to evaluate parathyroid function [11]. But the symptomatic hypocalcemia of group II were lower than group I, which indicated that the FOCUS may be helpful in protecting parathyroid.

Moreover, an important factor for the accurate assessment of the surgical time is the composition of the surgical team. In thyroid surgery, the surgeon usually dissects or applies the scalpel, leaving various surgical maneuvers to the assistant. So a good cooperation in the surgical team would allow for a further decrease in the operative time for both techniques.

According the study, the post-operative hospital stay was decreased by the new surgical technique.

## Conclusions

According to our results, the FOCUS Harmonic Scalpel can shorten operative time and hospital stay, reduce incidence of symptomatic hypocalcaemia but not transient hypoparathyroidism, and show no significance on recurrent nerve injury. FOCUS Harmonic Scalpel is supposed to be a more reliable and safe instrument that can take place of the clamp-and-tie technique in total thyroidectomy.

## Consent

Written informed consent was obtained from the patient for publication of this report and any accompanying images.

## Competing interests

The authors declare that they have no competing interests. No benefits in any form have been received or will be received from a commercial party related directly or indirectly to the subject of this article.

## Authors’ contributions

Y-fD, WX, and D-lS participated in the clinical management and wrote the manuscript. FZ participated in data analysis. Y-fD and D-lS were involved in the final editing. All authors approved the final manuscript.
